# A Cross-Scale Electrothermal Co-Simulation Approach for Power MOSFETs at Device–Package–Heatsink–Board Levels

**DOI:** 10.3390/mi15111336

**Published:** 2024-10-31

**Authors:** Yuxuan Dai, Jiafei Yao, Jing Chen, Qingyou Qian, Maolin Zhang, Jun Zhang, Qing Yao, Chenyang Huang, Mingshun Sun, Yufeng Guo

**Affiliations:** 1 College of Integrated Circuit Science and Engineering, Nanjing University of Posts and Telecommunications, Nanjing 210023, China; 2021020308@njupt.edu.cn (Y.D.); chenjing1992@njupt.edu.cn (J.C.); mlzhang@njupt.edu.cn (M.Z.); zhangjun1991@njupt.edu.cn (J.Z.); 2021020311@njupt.edu.cn (Q.Y.); 2020020115@njupt.edu.cn (C.H.); 2 National & Local Joint Engineering Laboratory for RF Integration and Micro-Packaging Technologies, Nanjing University of Posts and Telecommunications, Nanjing 210023, China; 3 JIEJIE Microelectronics Co., Ltd., Nantong 226200, China; qyqian@jjwdz.com (Q.Q.); smsun@jjwdz.com (M.S.)

**Keywords:** chip temperature, drain current, device–package–heatsink–board level, electrothermal co-simulation, power MOSFETs

## Abstract

This paper proposes a cross-scale simulation approach for evaluating the steady-state electrothermal performance of power MOSFETs at the device–package–heatsink–board (DPHB) level. A co-simulation framework is designed by employing the iterative process of power loss and chip temperature to bridge the device and package–heatsink–board (PHB) level simulators. As a result, the cross-scale electrothermal coupling effect within multilevel settings is considered. Correspondingly, variation values in chip temperature and temperature-dependent drain current can be obtained at various voltage biases, level settings, and DPHB structural parameters, incorporating cross-level physical insights. The simulation results are compared with existing methods, and their features and limitations are discussed. Additionally, this paper also derives an empirical equation from the co-simulations to characterize the relationship between the drain current and the chip temperature under different operations exactly. A commercial MOSFET with TO-220F packaging is implemented in experiments to extract the chip temperature and drain current in electrothermal equilibrium. The method comparisons and fair agreement among simulations, equations, and measurements presents the proposed approach as generalized and powerful for describing variations in chip temperature and drain current considering from micrometer devices to millimeter packages–heatsinks–PCB boards, thus providing effective support for DPHB-level co-design.

## 1. Introduction

Power MOSFETs are extensively employed in power control and conversion systems [1], where they are vulnerable to self-heating effects that elevate peak chip temperatures during operation [2]. The rise in chip temperature deteriorates the device’s electrical performance [3,4], leading to de-rating power output and failure to meet application requirements [5]. More seriously, excessive chip temperature can cause catastrophic damage to the device [6,7,8,9]. In view of device–package–heatsink–board (DPHB)-level structural parameters strongly affecting the device’s electrical performance and heat dissipation [10,11,12], an accurate assessment of the electrothermal performance during DPHB-level design stage can effectively avoid the aforementioned issues. However, there is vast scale variation ranging from micrometer-scale devices to millimeter-scale packages, heatsinks, and PCB boards. This complicates the electrothermal simulation at the DPHB level. The current electrothermal simulation methods can be categorized into three main types: single software simulation, direct coupling simulation, and iterative coupling simulation.

(1) Single software simulation. Commonly device-level simulator technology computer-aided design (TCAD) (e.g., Medici, Silvaco, Sentaurus) can comprehensively investigate electrothermal behavior in micrometer-scale devices. However, the packaging factors are usually simplified into a nominal boundary thermal resistance [13], with its value typically determined through experimental fitting. In contrast, typical package–heatsink–board (PHB)-level simulator finite element analysis (FEA) (e.g., ANSYS, COMSOL, FLOTHERM) can accurately simulate large-area heat flow, enabling complete millimeter-scale packages–heatsinks–boards to be considered. Due to the lack of device consideration [13], it fails to account for the thermal feedback on electrical performance. Current research suggests the direct integration of both device and package structures within the TCAD environment, establishing a connection between electrical and thermal simulations via thermal nodes [14,15,16]. The complexities of constructing sophisticated 3D structures in TCAD and its limitations in analyzing larger area heat flow compared to FEA, lead to the inadequate consideration of more types of PHB-level parameters and environmental factors.

(2) Direct coupling simulation. Some researchers proposed TCAD-FEA direct coupling simulation [13,17,18], which also aims to integrate packaging factors in TCAD. In this work, boundary thermal resistance is calculated through FEA simulations, eliminating the need for experimental determination. The method requires constructing the study structure in both TCAD and FEA, which may increase the tediousness of the simulation process. Additionally, the previously discussed limitations in accounting for more intricate 3D structures and environmental factors in TCAD are noted. Another prevalent method is embedding the equivalent thermal model into SPICE-like platforms [19,20,21,22]. The method necessitates rederiving the SPICE model when accounting for changes in device structural parameters and does not permit the observation of physical field distributions.

(3) Iterative coupling simulation. A common method involves iterative coupling with device models for electrical properties (e.g., physical models, behavioral models, look-up table models) and the equivalent thermal model for thermal properties [23,24,25,26,27]. However, similar to directly embedding thermal model into SPICE-like platforms, this method restricts the analysis of device structural parameter variations and physical field distributions.

In this paper, the TCAD-FEA iteration-based electrothermal co-simulation framework is established to evaluate variation values in chip temperature and the drain current of power MOSFETs at the DPHB level. The framework characterizes electrical behavior in the device-level simulator TCAD and thermal behavior in the PHB-level simulator FEA. Subsequently, these simulators are coupled together through an iterative process of power loss and chip temperature in order to achieve self-consistent electrothermal behavior. Distinguishing from existing methods, the established framework can comprehensively account for the effects of DPHB parameter variations, aiming to develop a generalized approach applicable to a wider range of DPHB structures and enabling integrated co-design optimization at the DPHB level.

## 2. Methodology

**Figure 1:**
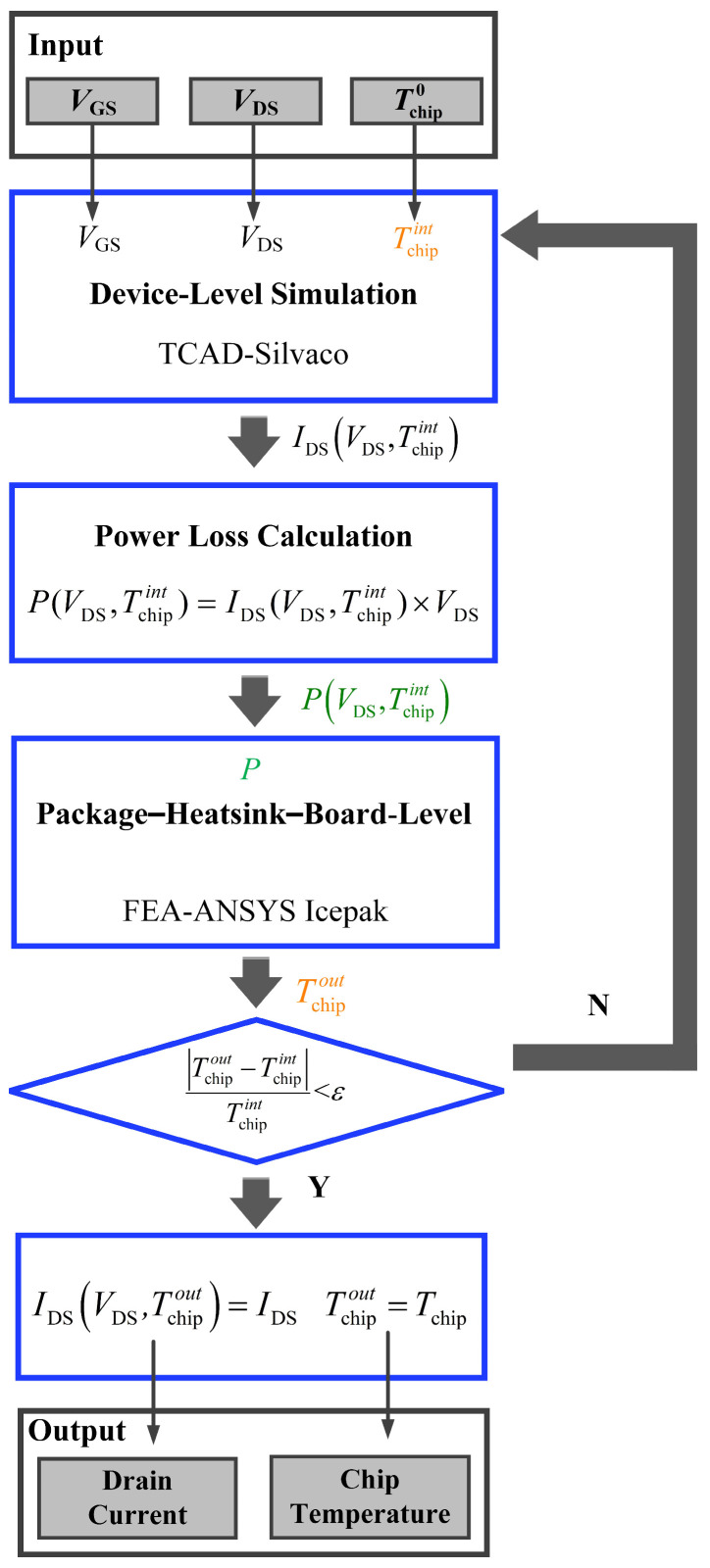
The iteration-based electrothermal co-simulation framework for evaluating chip temperature/drain current variations in power MOSFET at the DPHB level.

Figure 1 shows the proposed framework for evaluating power MOSFETs’ chip temperature/drain current variations at the DPHB level. In this paper, the device-level simulator Silvaco TCAD is utilized [28], while the PHB-level simulator employs ANSYS Icepak for FEA analysis. Synchronization and data transfer between Silvaco and ANSYS Icepak are provided by a Python script within the Linux environment. Firstly, determine the applied gate voltage bias (*V*_GS_), drain voltage bias (*V*_DS_), and initial ambient temperature ($T_{\mathrm{chip}}^{0}$) at which the device will be located (room temperature 25 °C for this paper). Based on the aforementioned conditions and the employed device structure, the output characteristics at the current chip temperature (*T*_chip_) are simulated in Silvaco. After that, the drain current (*I*_DS_) is recorded and the power loss (*P*) is calculated by:

(1) $$P(V_{\mathrm{DS}},T_{\mathrm{chip}})=I_{\mathrm{DS}}(V_{\mathrm{DS}},T_{\mathrm{chip}})\times V_{\mathrm{DS}}$$

The power loss is subsequently directly input into ANSYS Icepak. According to the employed package–heatsink–board structure and the determined initial ambient condition, ANSYS Icepak performs steady-state thermal simulations and obtains the updated chip temperature. Next, the updated chip temperature is compared with the previous one to assess the relative error. If the error falls below the predefined margin ε (defined as 1 × 10^3^ in this paper), the simulation iterative process is deemed complete. Meanwhile, the final drain current and chip temperature are output. The final iterations of temperature and current values represent the device’s actual steady-state chip temperature and drain current. Otherwise, the updated chip temperature is reintroduced into the device-level simulator Silvaco to repeat the iterative process.

**Figure 2:**
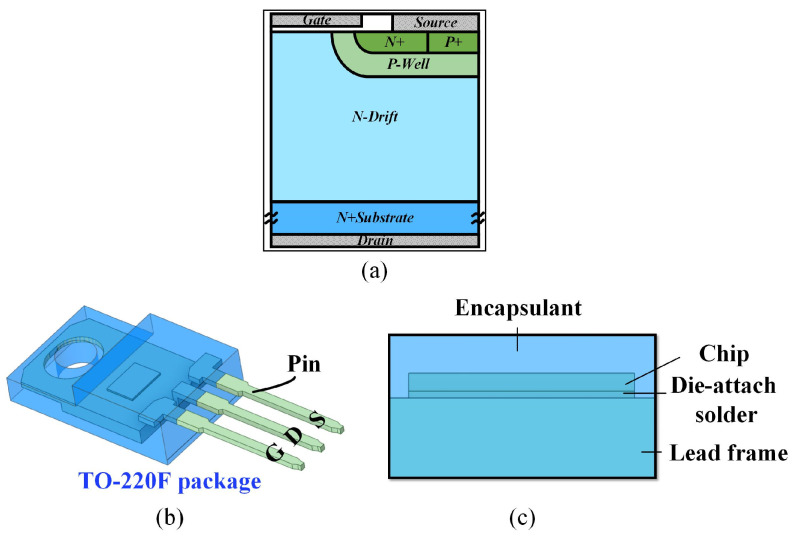
The schematic diagram of the researched commercial device. (**a**) Device-level structure, (**b**) package-level structure, and (**c**) cross-section.

A commercial power MOSFET with a rated voltage of 650 V, a rated current of 9 A, and a threshold voltage of 3 V is employed to validate the proposed approach, as illustrated in Figure 2. A comprehensive description of models utilized in Silvaco and simulation settings in ANSYS Icepak is provided for the selected commercial devices. The 2D micrometer-scale device structure (Figure 2a) is modeled using Silvaco, followed by electrical simulations. Since the operating conditions analyzed in this paper pertain to the on-state, the output characteristics are simulated using the following physical models. The Lombardi field-dependent mobility (*µ*_T_) model [29] is applied, with the parameter GAMN.CVT is modified from the default value of 2.5 to 2, and GAMP.CVT from 2.2 to 2, to better align the output characteristics with temperature variations. The main equation of the model is:

(2) $$\frac{1}{\mu_{T}}=\frac{1}{\mu_{\mathrm{AC}}}+\frac{1}{\mu_{b}}+\frac{1}{\mu_{\mathrm{sr}}}$$

Here, *µ*_AC_ is the surface mobility, *µ*_b_ is the mobility limited by scattering with optical intervalley phonons and *µ*_sr_ is the surface roughness factor. Additionally, the Fermi–Dirac statistics model [30] is also employed, given the main equation below:

(3) $$f(\varepsilon )=\frac{1}{1+\exp(\frac{\varepsilon -E_{F}}{kT_{L}})}$$

Here, *ε* is the energy, *f*(*ε*) is the probability that an available electron state with *ε*, *E*_F_ is a spatially independent reference energy known as the Fermi level, *k* is Boltzmann’s constant, and *T*_L_ is the lattice temperature. Moreover, the Shockley–Read–Hall model [31,32] is added and the primally equation is given:

(4) $$R_{\mathrm{SRH}}=\frac{pn-n_{\mathrm{ie}}^{2}}{\mathrm{TAUP}0\left[n+n_{\mathrm{ie}}exp(\frac{\mathrm{ETRAP}}{kT_{L}})\right]+\mathrm{TAUN}0\left[p+n_{\mathrm{ie}}exp(\frac{-\mathrm{ETRAP}}{kT_{L}})\right]}$$

**Figure 3:**
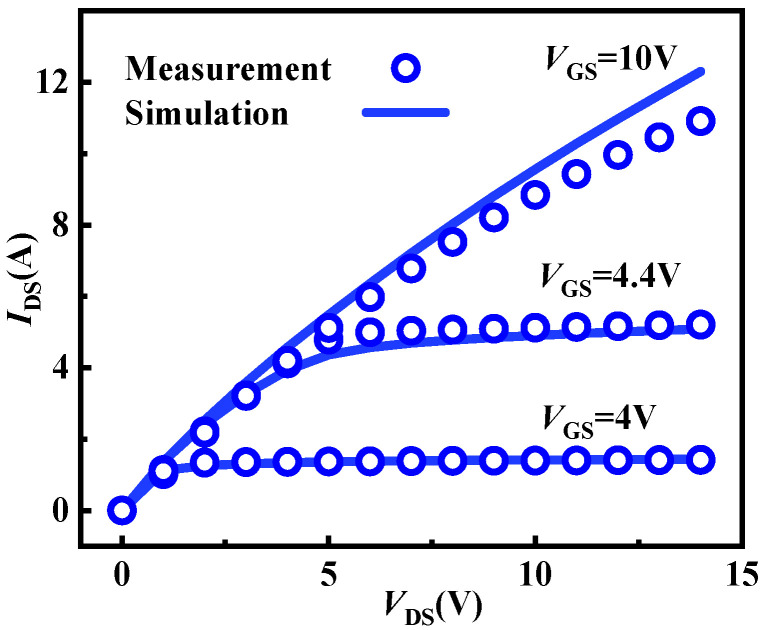
Comparisons of output characteristics at 25 °C in measurements and simulations for the researched commercial device.

Here, *R*_SRH_ is the Shockley–Read–Hall recombination, *n* and *p* are carrier concentrations, *n*_ie_ is intrinsic carrier concentration, TAUP0 and TAUN0 are the electron and hole lifetimes, and ETRAP is the difference between the trap energy level and the intrinsic Fermi level. In addition, the interface state is set as 3 × 10^10^ cm^−2^. The gate width (third dimension) for the 2D simulation is substituted with a coefficient area factor of 3 × 10^6^ μm. The output characteristics of the commercial device, obtained from measurements and Silvaco simulations, are presented in Figure 3, demonstrating substantial consistency. The millimeter-scale package structure (Figure 2b) is modeled and imported into ANSYS Icepak to thermal simulation. The key parameters of the device’s package structure are listed in Table 1. While the wire bonds in the package structure have a low impact on heat dissipation, this paper ignores them to reduce the simulation complexity. Based on the working conditions, the thermal simulation is conducted under natural convection settings.

**Figure 4:**
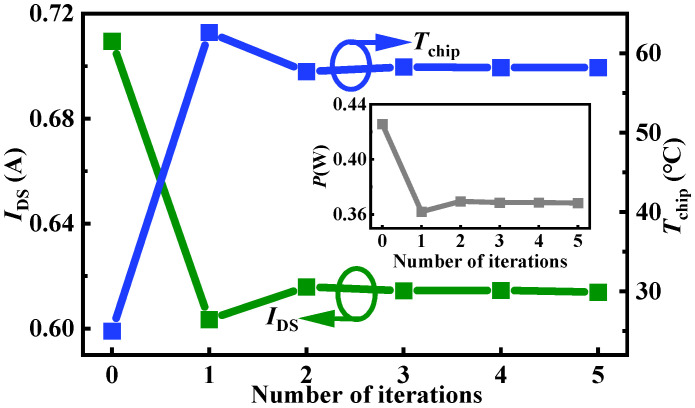
The drain current (green line), chip temperature (blue line), and power loss (gray line) with the number of iterations in DP-level co-simulation under *V*_GS_ = 10 V and *V*_DS_ = 0.6 V.

**Figure 5:**
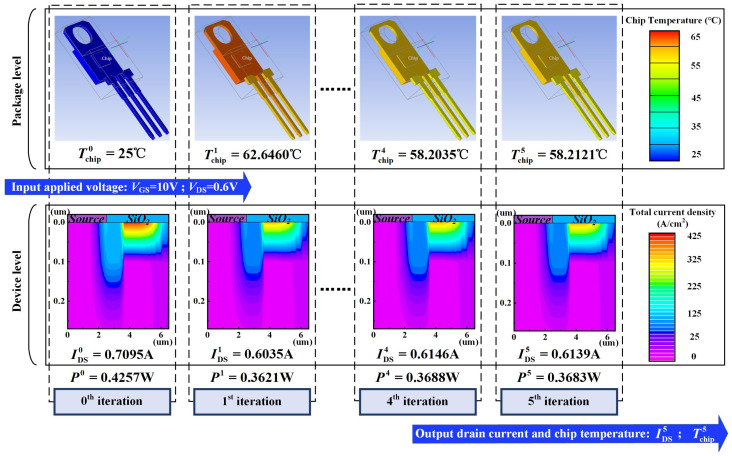
The ANSYS Icepak simulated chip temperature distribution at the package level and Silvaco simulated current density distribution at the device level in each iteration under *V*_GS_ = 10 V and *V*_DS_ = 0.6 V.

Figure 4 and Figure 5 give a sample of the chip temperature and drain current variations simulation based on the proposed approach. Figure 4 shows the drain current, chip temperature, and power loss with the number of iterations in device-package (DP) level co-simulation under *V*_GS_ = 10 V and *V*_DS_ = 0.6 V. Figure 5 shows ANSYS Icepak-simulated chip temperature distribution at the package level, and Silvaco-simulated current density distribution at the device level in each iteration. From the simulation results, it can be observed that the drain current begins to flow within the device after applying voltage bias. Subsequently, the power loss can be calculated and input into ANSYS Icepak, leading to an increase in chip temperature. While the increased chip temperature input into Silvaco further impacts the drain current, an interaction exists between drain current, power loss, and chip temperature. As the number of iterations increases, the changes in these values gradually diminish and reach the *ε* setting at the 5th iteration. In this sample, the chip temperature rises by 33 °C, and the drain current drops by 13%.

## 3. Simulation Results and Analysis

### 3.1. Device–Package-Level Co-Simulation

To investigate the effect of different operations on the chip temperature and drain current variations, DP level co-simulations are conducted by utilizing the proposed approach.

**Figure 6:**
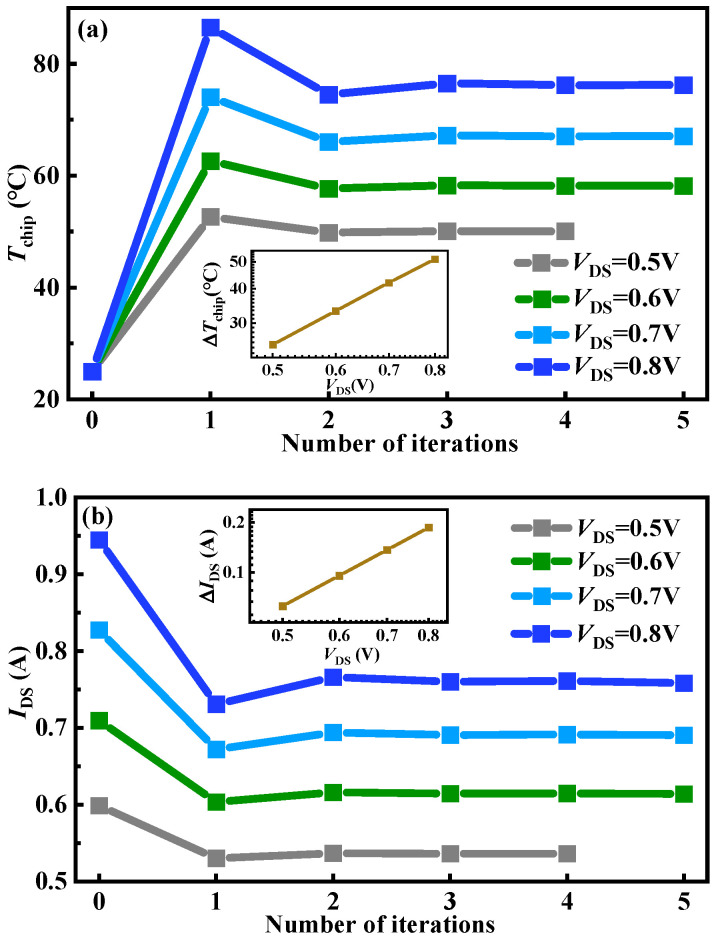
The chip temperatures and drain currents with the number of iterations in DP-level co-simulations under *V*_GS_ = 10 V and various *V*_DS_. (**a**) The chip temperatures change with the number of iterations, and (**b**) the drain currents change with the number of iterations.

**Figure 7:**
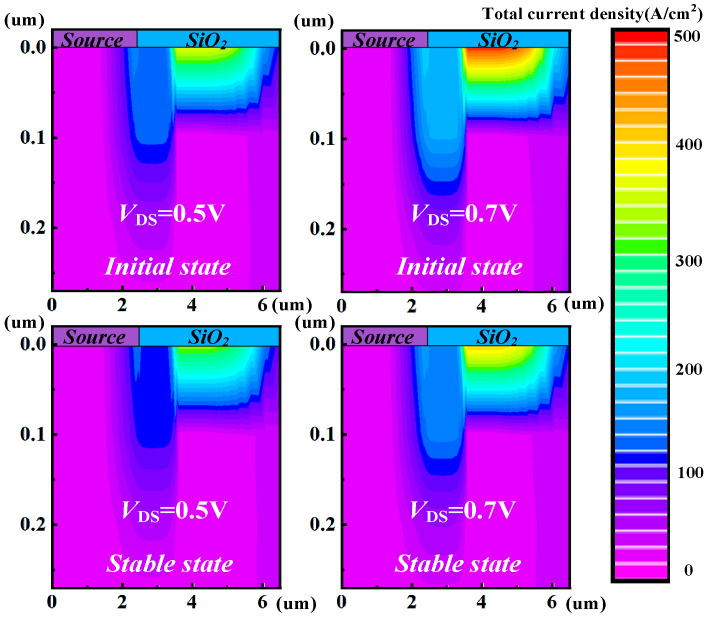
The initial and stable states of current density distributions under *V*_GS_ = 10 V and *V*_DS_ = 0.5 V and *V*_GS_ = 10 V and *V*_DS_ = 0.7 V, respectively.

Figure 6 shows the chip temperatures and drain currents with the number of iterations in DP-level co-simulations under *V*_GS_ = 10 V and various *V*_DS_. In addition, the subplot in Figure 6 shows the chip temperature and drain current variations with the applied *V*_DS_ in double logarithmic coordinates, which satisfies the following proposed mathematical fitting equation, respectively:

(5) $$\Delta T_{\mathrm{chip}}=T_{\mathrm{chip}}-T_{\mathrm{chip}}^{0}=C_{1}\times V_{\mathrm{DS}}^{\beta_{1}}$$

(6) $$\Delta I_{\mathrm{DS}}=I_{\mathrm{DS}}^{0}-I_{\mathrm{DS}}=C_{2}\times V_{\mathrm{DS}}^{\beta_{2}}$$

where the $I_{\mathrm{DS}}^{0}$ is the initial state of the drain current. Moreover, the parameters of *C*_1_ and *C*_2_ are the fitting coefficient constants. The parameters of *β*_1_ and *β*_2_ are fit exponents. They donate the extent of chip temperature/drain current variation with the applied drain voltage bias. After fitting, *C*_1_ is 72.02, *C*_2_ is 0.31, *β*_1_ is 1.52, and *β*_2_ is 2.32. The simulation results indicate that the larger *V*_DS_ leads to a marked variation in chip temperature and drain current. This phenomenon is primarily attributed to the rise in chip temperature, which heightens the scattering probability of carriers. Consequently, carriers encounter more obstacles while traversing the semiconductor material, thereby diminishing their mobility [33]. The reduced mobility subsequently elevates the device on-resistance [34], leading to a decay in drain current. The higher chip temperature is induced by the increased power consumption due to the larger *V*_DS_. Thus, further reducing mobility and increasing on-resistance exacerbating the variation in drain current. Figure 7 shows the initial and stable states of the current density distribution under *V*_GS_ = 10 V and *V*_DS_ = 0.5 V and *V*_GS_ = 10 V and *V*_DS_ = 0.7 V, respectively. It can be clearly observed that the smaller *V*_DS_ has a relatively minor impact on the change in current density.

### 3.2. Device–Package–Heatsink–Board-Level Co-Simulation

In this section, the effect of different level settings on the chip temperature and drain current variations is investigated. The level settings include the DP level, the device–package–heatsink (DPH) level, and the DPHB level. It is important to note that since the circuit is not considered in this paper, PCB lines and layout have been excluded, with the primary focus placed on the thermal conductivity of the PCB material. The key parameters of the heatsink and PCB board are listed in Table 2.

**Figure 8:**
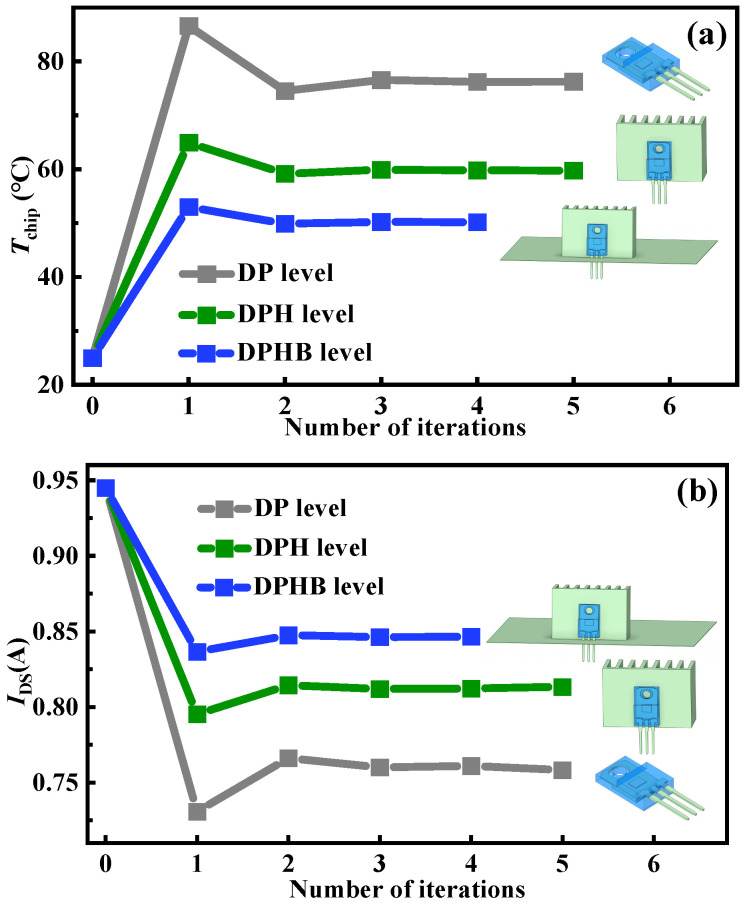
The chip temperatures and drain currents with the number of iterations under *V*_GS_ = 10 V and *V*_DS_ = 0.8 V at various level settings. (**a**) The chip temperatures change with the number of iterations, and (**b**) the drain currents change with the number of iterations.

**Figure 9:**
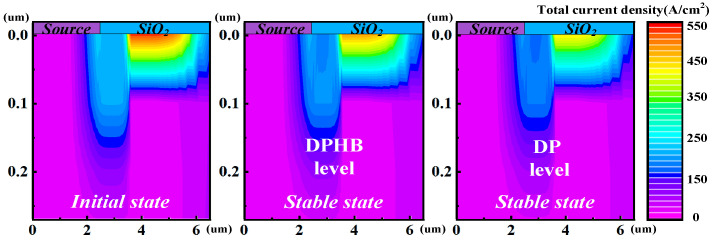
The initial and stable states of current density distributions under *V*_GS_ = 10 V and *V*_DS_ = 0.8 V at the DP level and DPHB level, respectively.

Figure 8 shows the chip temperatures and drain currents with the number of iterations under *V*_GS_ = 10 V and *V*_DS_ = 0.8 V at different level settings. The simulation results reveal the maximum variations in drain current and chip temperature occur when the heatsink–PCB board is not utilized. However, with the implementation of the heatsink alone, drain current variation decreases by 6% and the maximum chip temperature has a reduction of 17 °C. Using both the heatsink and PCB board further reduces drain current variation by 9%, with a maximum chip temperature decrease of 26 °C. The main reason for this is that the heatsink and PCB board expedite temperature dissipation in the device, diminishing the impact of the temperature field effect. Figure 9 shows the initial and stable states of the current density distribution under *V*_GS_ = 10 V and *V*_DS_ = 0.8 V at the DP level and DPHB level, respectively. It can be observed that the influences of both the heatsink and PCB board on the change in current density are relatively minor.

### 3.3. Device–Package–Heatsink–Board-Level Parameter Analysis

**Figure 10:**
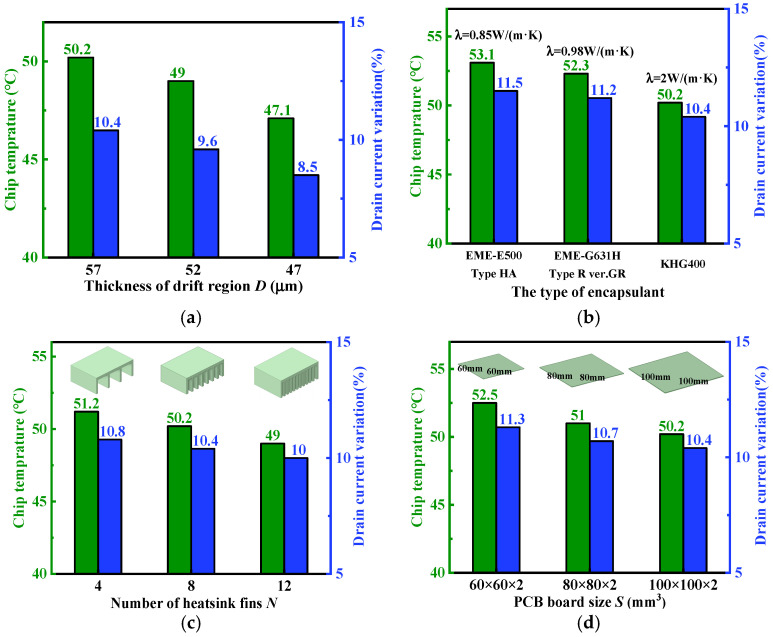
Device–package–heatsink–board-level parameters versus maximum chip temperature and drain current variation. (**a**) The change in device-level parameter *D*, where *λ* is 2 W/(m·K), *N* is 8, and *S* is 100 × 100 × 2 mm^3^; (**b**) the change in package-level parameter *λ*, where *D* is 57 µm, *N* is 8, and *S* is 100 × 100 × 2 mm^3^; (**c**) the change in heatsink-level parameter *N*, where *D* is 57 µm, *λ* is 2 W/(m·K), and *S* is 100 × 100 × 2 mm^3^; (**d**) the change in board-level parameter *S*, where *D* is 57 µm, *λ* is 2 W/(m·K), and *N* is 8.

In this section, the impacts of DPHB-level structural parameters on chip temperature and drain current are shown in Figure 10. For the device-level parameter, the drift region thickness *D* is investigated. A change in *D* affects the basic electrical characteristics of the device. The approach not only evaluates the fundamental electrical characteristics from different device structural parameters but also calculates the electrothermal performance during operation. For the package-level parameter, the encapsulant thermal conductivity *λ* is investigated. Three types of encapsulants, EME-E500 Type HA, EME-G631H Type R ver.GR, and KHG400, are selected for simulation. Due to material development constraints, an increase in *λ* does not have a significant impact in an open area. For heatsink–board-level parameters, the heatsink fins number *N* and the PCB board size *S* are investigated. There exists a trade-off between size and performance enhancement. It is evident that increasing *N* and *S* has limited effects.

## 4. Experimental

The researched commercial device, a heatsink measuring 25 × 36 × 16 mm^3^ with 8 fins, and a PCB board measuring 100 × 100 × 2 mm^3^ without lines and layout are utilized to conduct a series of tests.

### 4.1. The Variation in Drain Current

**Figure 11:**
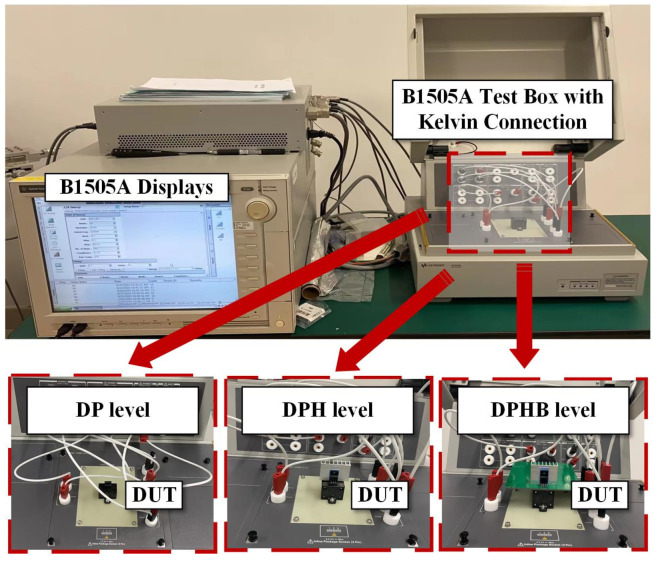
Experimental setup (Agilent B1505A without heating platform).

**Figure 12:**
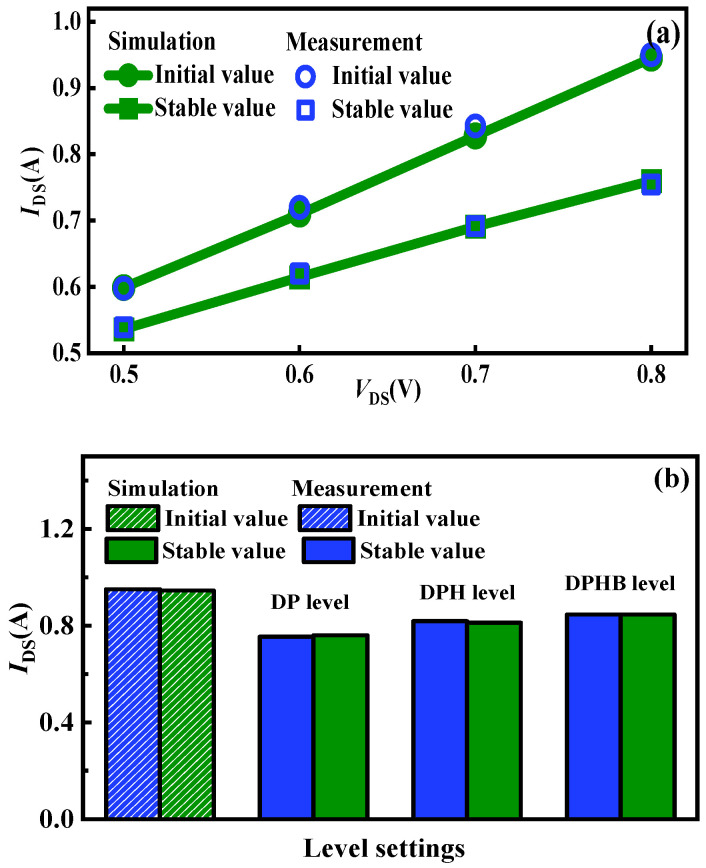
(**a**) The initial and stable states of drain currents in measurements and simulations under *V*_GS_ = 10 V and various *V*_DS_. (**b**) The initial and stable states of drain currents in measurements and simulations under *V*_GS_ = 10 V and *V*_DS_ = 0.8 V at various level settings.

To verify the accuracy of the simulated drain current variation values from initial to steady state, a semiconductor parameter analyzer (Keysight Agilent B1505A, Santa Rosa, CA, USA) is utilized, as shown in Figure 11. All the tests are conducted at room temperature. The device under test (DUT) is placed inside the test box of the Agilent B1505A with a Kelvin connection to measure the drain current over time. Then, the drain current that changed to a stable value is recorded. A note regarding the limited voltage range utilized to characterize the devices: this paper aims to focus on validating the self-heating modeling within the device, which can be easier achieved by maintaining a low *V*_DS_ to avoid exceeding the limitations of the source measurement unit (SMU) [26]. The initial and stable states of drain currents in measurements and simulations are shown in Figure 12. It can be observed that the drain current variations under diverse operations and level settings can be accurately reflected by the proposed approach, with a margin of error within 1%.

### 4.2. The Variation in Chip Temperature

**Figure 13:**
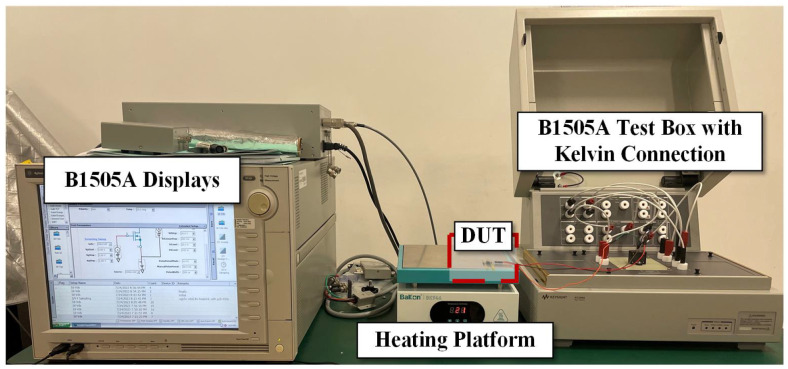
Experimental setup (Agilent B1505A with heating platform).

**Figure 14:**
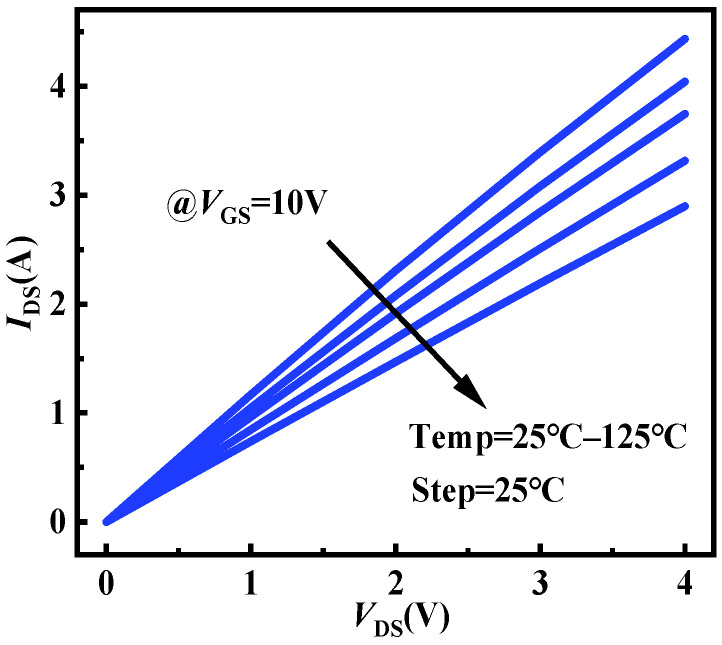
The device’s output characteristics under *V*_GS_ = 10 V at different temperatures.

**Figure 15:**
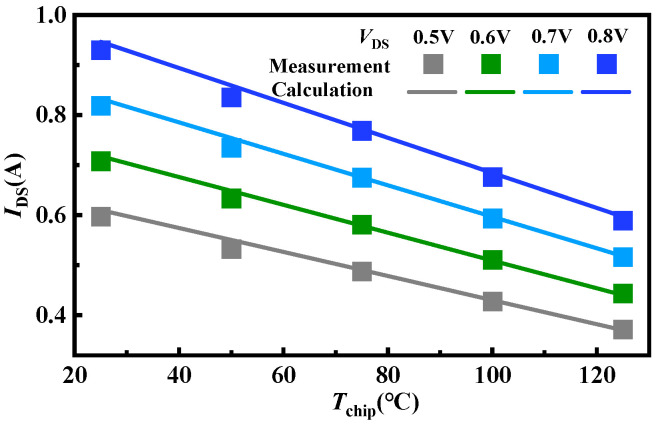
Relationships between drain currents and chip temperatures under *V*_GS_ = 10 V and various *V*_DS_.

**Figure 16:**
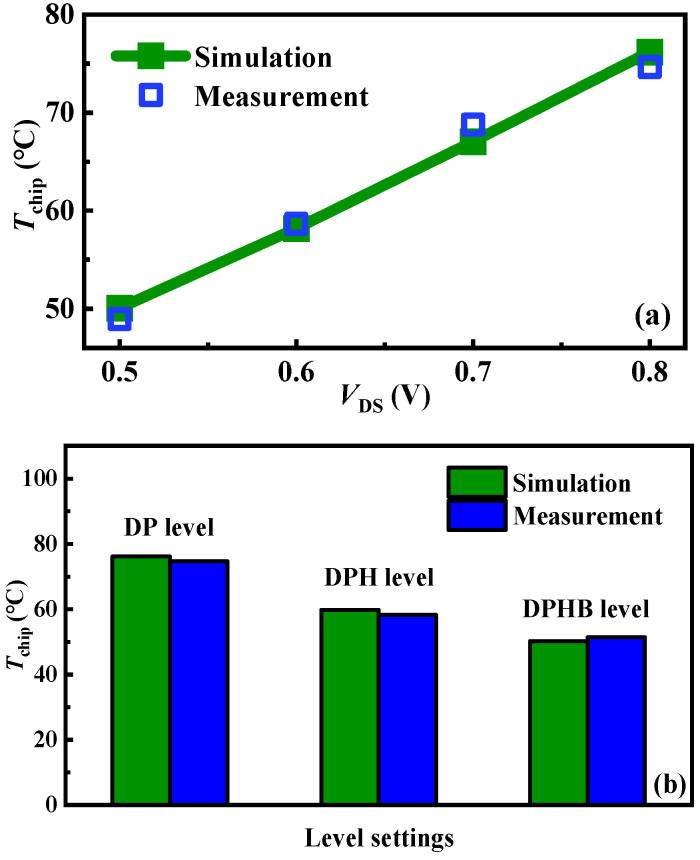
(**a**) The stable states of chip temperatures in measurements and simulations under *V*_GS_ = 10 V and various *V*_DS_. (**b**) The stable states of chip temperatures in measurements and simulations under *V*_GS_ = 10 V and *V*_DS_ = 0.8 V at various level settings.

To validate the accuracy of the simulated chip temperature variation values from initial to steady state, an indirect calculation from the drain current values is attempted. An experimental setup is established to measure the drain current at different temperatures, as shown in Figure 13. The setup consists of an Agilent B1505A and a heating platform. The DUT is placed on the heating platform and introduced into the test box of the Agilent B1505A by electrical connections. The device’s drain current characteristics under *V*_GS_ = 10 V at different temperatures are measured, as shown in Figure 14. Subsequently, the drain currents at temperature ranges can be extracted from the different *V*_DS_ values (Figure 15 symbols). It is worth noting that according to Equations (5) and (6), the following empirical equation is obtained:

(7) $$I_{\mathrm{DS}}=-CV_{\mathrm{DS}}^{\beta }\times T_{\mathrm{chip}}+CV_{\mathrm{DS}}^{\beta }\times T_{\mathrm{chip}}^{0}+I_{\mathrm{DS}}^{0}$$

where *C* = *C*_2_/*C*_1_ = 0.0043 and *β* = *β*_2_ − *β*_1_ = 0.8. Based on (7), the relationships between drain currents and chip temperatures with *V*_DS_ can be calculated (Figure 15 lines). The relationship in measurements and calculations exhibits excellent consistency and linearity. Consequently, the chip temperature is calculated through the drain current. As the tests are conducted at room temperature, the initial temperature for all examples is identical to ambient conditions. A comparison of steady-state chip temperatures from both measurements and simulations is presented in Figure 16. It can be seen that the chip temperature variation under diverse operations and level settings can be accurately obtained by the proposed approach, with a margin of error of 3%.

### 4.3. Comparison of Methods

**Figure 17:**
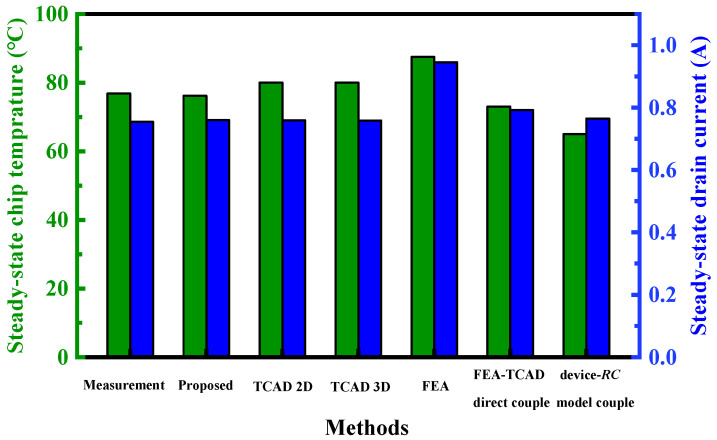
Comparison of existing methods: assessment of steady-state chip temperature (green bars) and drain current (blue bars) under *V*_GS_ = 10 V and *V*_DS_ = 0.8 V.

The proposed approach is compared with single software simulations and direct coupling simulations, as well as other methods in iterative coupled simulations, as displayed in Figure 17. For single software simulations, TCAD (Sentaurus) and FEA (ANSYS Icepak) simulations are employed. In TCAD 2D and 3D simulations, the device structure shown in Figure 2a is modeled, and the packaging factor is simplified into substrate boundary thermal resistance, which is adjusted through measurement fitting. In FEA simulations, variations in chip temperature cannot be fed back into the electrical performance due to the inability to consider the device’s behavior. Consequently, the results of this method differ significantly from the measurement. For the direct coupling simulation, TCAD-FEA direct simulation is selected, following the steps outlined in [13,17]. First, the 3D package structure needs to be modeled in both TCAD and FEA. Since constructing complex structures in TCAD is more challenging, only models the lead frame component in this work, which has a greater impact on heat dissipation. Next, the boundary thermal resistance of the device to the lead frame and the bottom of the lead frame is calculated from the FEA simulation results, rather than being determined by constant fitting based on measurements. Discrepancies with measurement data may arise from the fact that the complete package structure is not considered in TCAD. For another method in iterative coupled simulation, the device model is coupled with the *RC* Cauer thermal model. The device model selected the on-resistance model to calculate the variation of device electrical performance with temperature, which results from temperature-dependent mobility and intrinsic carrier concentration [34]. These two models are iterative coupling and finally obtain the steady-state electrothermal parameters.

## 5. Conclusions

This paper presents a cross-scale co-simulation approach for evaluating steady-state electrothermal performance in power MOSFETs at the device–package–heatsink–board (DPHB) level. By iteratively combining power loss and chip temperature through Python script, the device-level simulator Silvaco is integrated with the package–heatsink–board (PHB)-level simulator ANSYS Icepak, forming a comprehensive co-simulation framework. This framework enables the consideration of cross-scale electrothermal behavior to adapt to different operating conditions, multilevel settings, and DPHB-level structural parameters. Moreover, from simulation results, an empirical equation is derived to characterize the relationship between the drain current and the chip temperature. Finally, the simulation results are compared with the measured data and results of other methods. The approach considers complete DPHB-level structural parameters and also shows potential for broader applications across various device–package–heatsink–board types, facilitating comprehensive DPHB co-design. While this paper emphasizes steady-state analysis under static conditions, it is anticipated to extend to transient analysis and dynamic switching conditions in future work.

## Figures and Tables

**Table 1:** Key parameters of the package-level structure.

Layer	Geometric Size [mm^3^]	Thermal Material	Thermal Conductivity [W/(m·K)]
Chip	4.1 × 3.1 × 0.16	Si	148
Die-attach solder	4.1 × 3.1 × 0.06	Pb_92.5_Sn_5_Ag_2.5_	26
Lead frame	/	Cu	387
Pin	/	Cu	387
Encapsulant	/	Epoxy resin	2

**Table 2:** Key parameters of the heatsink and PCB board.

Level Setting	Geometric Size [mm^3^]	Thermal Material	Thermal Conductivity [W/(m·K)]
Heatsink	25 × 36 × 16	Al	205
PCB board	100 × 100 × 2	Cu/FR-4	387/0.35

## Data Availability

The original contributions presented in the study are included in the article; further inquiries can be directed to the corresponding authors.
